# Machine learning based on Optical Coherence Tomography images as a diagnostic tool for Alzheimer's disease

**DOI:** 10.1111/cns.13963

**Published:** 2022-09-11

**Authors:** Xin Wang, Bin Jiao, Hui Liu, Yaqin Wang, Xiaoli Hao, Yuan Zhu, Bei Xu, Huizhuo Xu, Sizhe Zhang, Xiaoliang Jia, Qian Xu, Xinxin Liao, Yafang Zhou, Hong Jiang, Junling Wang, Jifeng Guo, Xinxiang Yan, Beisha Tang, Rongchang Zhao, Lu Shen

**Affiliations:** ^1^ Department of Neurology, Xiangya Hospital Central South University Changsha China; ^2^ National Clinical Research Center for Geriatric Disorders Central South University Changsha China; ^3^ Engineering Research Center of Hunan Province in Cognitive Impairment Disorders Central South University Changsha China; ^4^ Hunan International Scientific and Technological Cooperation Base of Neurodegenerative and Neurogenetic Diseases Changsha China; ^5^ Key Laboratory of Hunan Province in Neurodegenerative Disorders Central South University Changsha China; ^6^ Health Management Center, the Third Xiangya Hospital Central South University Changsha China; ^7^ Eye Center of Xiangya Hospital Central South University Changsha China; ^8^ School of Computer Science and Engineering Central South University Changsha China; ^9^ Department of Geriatrics, Xiangya Hospital Central South University Changsha China; ^10^ Key Laboratory of Organ Injury Aging and Regenerative Medicine of Hunan Province Changsha China

**Keywords:** Alzheimer's disease, diagnosis, machine learning, optical coherence tomography, retina

## Abstract

**Aims:**

We mainly evaluate retinal alterations in Alzheimer's disease (AD) patients, investigate the associations between retinal changes with AD biomarkers, and explore an optimal machine learning (ML) model for AD diagnosis based on retinal thickness.

**Methods:**

A total of 159 AD patients and 299 healthy controls were enrolled. The retinal parameters of each participant were measured using optical coherence tomography (OCT). Additionally, cognitive impairment severity, brain atrophy, and cerebrospinal fluid (CSF) biomarkers were measured in AD patients.

**Results:**

AD patients demonstrated a significant decrease in the average, superior, and inferior quadrant peripapillary retinal nerve fiber layer, macular retinal nerve fiber layer, ganglion cell layer (GCL), inner plexiform layer (IPL) thicknesses, as well as total macular volume (TMV) (all *p* < 0.05). Moreover, TMV was positively associated with Mini‐Mental State Examination and Montreal Cognitive Assessment scores, IPL thickness was correlated negatively with the medial temporal lobe atrophy score, and the GCL thickness was positively correlated with CSF Aβ_42_/Aβ_40_ and negatively associated with p‐tau level. Based on the significantly decreased OCT variables between both groups, the XGBoost algorithm exhibited the best diagnostic performance for AD, whose four references, including accuracy, area under the curve, f1 score, and recall, ranged from 0.69 to 0.74. Moreover, the macular retinal thickness exhibited an absolute superiority for AD diagnosis compared with other enrolled variables in all ML models.

**Conclusion:**

We identified the retinal alterations in AD patients and found that macular thickness and volume were associated with AD severity and biomarkers. Furthermore, we confirmed that OCT combined with ML could serve as a potential diagnostic tool for AD.

## Graphical abstract

In this study, we found that AD patients demonstrated a significant decrease in peripapillary retinal nerve fiber layer thickness and macular retinal thickness, as well as total macular volume. In addition, these changes were correlated with MRI findings indicative of cerebral atrophy, tau and amyloid‐beta levels in the cerebrospinal fluid, and *APOE* genotype. Finally, we found the XGBoost algorithm exhibited the best diagnostic performance for AD.

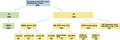

## 1 INTRODUCTION

Alzheimer's disease (AD) is the most common neurodegenerative disease and is the leading cause of dementia. It is pathologically characterized by extraneuronal amyloid‐β (Aβ) plaque deposition and intracellular neurofibrillary tangles consisting of hyperphosphorylated microtubule‐associated protein tau or tau.^1^ It has been reported that the prevalence of AD continues to rise worldwide, posing a serious burden on societies and their families.^2^ Studies have suggested that changes in positron emission tomography (PET) and cerebrospinal fluid (CSF) biomarkers can be detected years before the emergence of symptoms.^3^ Currently, the diagnosis of AD is reliant on detecting decreased Aβ_42_ and elevated tau levels in the CSF,^4^ cerebral glucose hypometabolism, and increased Aβ and tau deposition on PET images.^5^ However, these modalities are invasive, time‐consuming, and expensive; therefore, impractical for mass screening for AD. Thus, to achieve early diagnosis and intervention, it is necessary to develop a rapid, noninvasive, and straightforward tool to identify AD.

The retina might be an important site of inquiry because it shares the same embryonic precursor as the brain and displays several structural and functional similarities with the organ.^6^ Previous research suggests that visual symptoms, including impaired visual acuity, visual field, motion perception, and stereopsis, are frequently early symptoms of AD.^7^ In the past, these symptoms have been attributed to degeneration of the central visual pathways. However, recent studies have shown that the retina is also involved.^8^, ^9^ Additionally, Aβ deposits have been found in the retina of AD patients and mouse model.^10^

High‐definition optical coherence tomography (HD‐OCT) is a cost‐effective and convenient tool that allows for high‐resolution retinal images to be obtained in vivo. With the advent of OCT, the retina has become an appealing subject in many studies on neurodegenerative diseases, including AD.^11^, ^12^, ^13^, ^14^, ^15^, ^16^, ^17^ However, previous OCT findings are controversial; some studies have reported a thinning of the retina in AD,^11^, ^12^, ^13^, ^14^ whereas others have observed no change.^15^, ^16^, ^17^ While there has been much interest in exploring the potential correlation between dementia and retinal parameters, there is a paucity of comprehensive and consistent data from currently available studies.

Artificial intelligence (AI), particularly machine learning, has recently emerged as a powerful tool in the field of neuroscience. It refers to the ability of computers to detect complex patterns and construct models by learning from existing data. Machine learning has been developed and applied extensively in the field of medicine, including disease diagnosis and prognosis, disease progression monitoring, and treatment efficacy evaluation.^18^, ^19^, ^20^ Therefore, in this study, we first evaluated retinal alterations in patients with AD and then assessed the relationships between the retinal structure and the clinical parameters, as well as AD biomarkers. Finally, we evaluated the performance of six common machine learning algorithms based on OCT images.

## 2 METHODS

### 2.1 Study population

**FIGURE 1:**
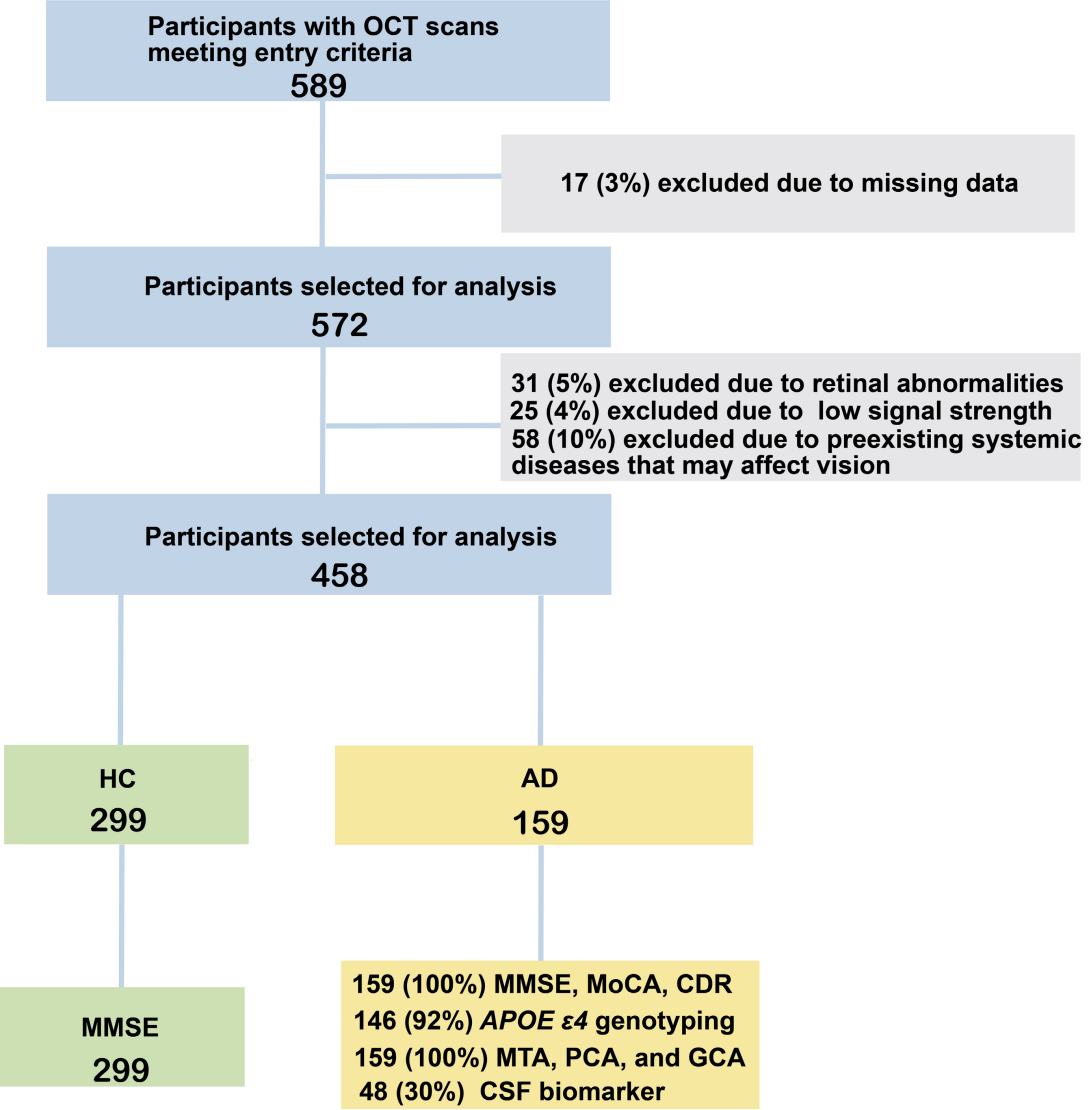
Flow chart of participants recruitment into the study. A total of 159 patients with AD and 299 age‐ and sex‐matched HCs were included in the final analysis. AD, Alzheimer's disease; HCs, healthy controls.

A total of 159 patients with AD and 299 healthy controls (HCs) were enrolled between December 2017 and November 2019. Patients with AD were recruited from Xiangya Hospital, and HCs were referred from the Health Management Center of the Third Xiangya Hospital. All diagnoses of probable or possible AD were performed by neurologists at Xiangya Hospital, based on the National Institute on Aging‐Alzheimer's Association guidelines.^21^ All participants underwent a complete ophthalmic examination and neuropsychological assessment. Patients with AD additionally underwent magnetic resonance imaging (MRI) of the brain, CSF biomarker testing, and apolipoprotein E (*APOE*) genotyping. Patients with (1) best‐corrected visual acuity (BCVA) <0.5, (2) refractive spherical equivalent >6.00 D or with astigmatism >3.00 D, and (3) intraocular pressure (IOP) >21 mmHg were excluded. All participants were free of diabetes mellitus and uncontrolled hypertension, as well as other neurologic, psychiatric, and ophthalmological conditions (including cataract, glaucoma, uveitis, epiretinal membrane, macular hole, age‐related macular degeneration, history of eye trauma, and any eye surgery). Figure 1 shows a flowchart for participants’ recruitment. Informed consent was provided by all participants or a first‐degree relative. All procedures followed the Declaration of Helsinki and were approved by the ethics committee of Xiangya Hospital (approval no. 201811199).

### 2.2 HD‐OCT examination

**FIGURE 2:**
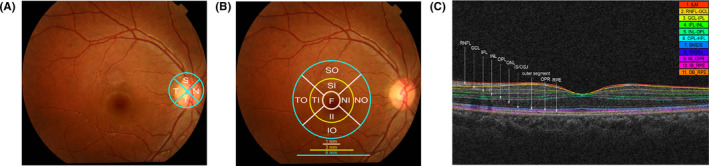
The retinal OCT scan protocol. (A), The four quadrants (superior, inferior, nasal, and temporal) pRNFL thickness measurement; (B), The nine ETDRS macular subfields map (SO, SI, II, IO, TO, TI, NI, NO, F); (C), The cross‐sectional OCT image. From the internal limiting membrane to the retinal pigment epithelium, the retina was segmented into the following 10 layers: mRNFL, GCL, IPL, INL, OPL, ONL, IS/OSJ, outer segment, OPR, and RPE. OCT, optical coherence tomography; pRNFL, peripapillary retinal nerve fiber layer; ETDRS, Early Treatment Diabetic Retinopathy Study; SO, superior outer; SI, superior inner; II, inferior inner; IO, inferior outer; TO, temporal outer; TI, temporal inner; NI, nasal inner; NO, nasal outer; F, fovea; mRNFL, retinal nerve fiber layer; GCL, ganglion cell layer; IPL, inner plexiform layer; INL, inner nuclear layer; OPL, outer plexiform layer; ONL, outer nuclear layer; IS/OSJ, inner segments/outer segments junction; OPR, outer segment photoreceptor/retinal pigment epithelium junction; RPE, retinal pigment epithelium layer.

All OCT scans (Cirrus HD‐OCT 4000; Carl Zeiss Meditec, Dublin, California) were performed by the same ophthalmologist, who was blinded to the diagnoses according to standard protocols. Due to time constraints and a high inter‐eye correlation between both eyes, we selected one random eye for the HD‐OCT examination. The thickness of the peripapillary retinal nerve fiber layer (pRNFL) was acquired by taking three consecutive 3.4‐mm circular scans centered on the optic nerve disk (Figure 2A). The macular parameters were measured by taking six consecutive 6‐mm radial line scans, each containing 128 A‐scans within a 6 × 6 mm macular region. The macular retinal thickness (MRT) was measured in nine regions corresponding to the procedures of the Early Treatment Diabetic Retinopathy Study (ETDRS) (Figure 2B). The pRNFL thickness (mean, superior, inferior, nasal, and temporal), MRT averaged over all nine ETDRS subfields, and total macular volume (TMV) were analyzed in this study. We excluded participants with HD‐OCT images of poor quality (signal strength <7) and those who could not follow OCT procedures due to severe cognitive impairment.

### 2.3 Macular intraretinal layer segmentation

All OCT images were segmented using advanced automated three‐dimensional retinal layer segmentation software (IOWA OCTExplorer v3.8.0).^22^ Segmentation was performed by two independent computer engineers who were blinded to the diagnosis of the participants. Briefly, this algorithm outlined 11 optical surfaces, from the internal limiting membrane to the retinal pigment epithelium, and automatically segmented the retina into the following 10 layers: retinal nerve fiber layer (mRNFL), ganglion cell layer (GCL), inner plexiform layer (IPL), inner nuclear layer, outer plexiform layer, outer nuclear layer, inner segments/outer segments junction, outer segment, outer segment photoreceptor/retinal pigment epithelium junction, and retinal pigment epithelium layer (Figure 2C). The thickness of the intraretinal layer was calculated for each of the nine ETDRS subfields. The average macular intraretinal layer thickness of the nine ETDRS grids was then calculated for further analysis.

### 2.4 Magnetic resonance imaging and CSF biomarkers

All patients underwent 3‐Tesla MRI scans on the same scanner (Magnetom Verio; Siemens, Erlangen, Germany). The severity of brain atrophy was assessed using three rating scales: Medial temporal lobe atrophy (MTA),^23^ global cortical atrophy (GCA),^24^ and Koedam's scale for parietal cortical atrophy (PCA).^25^ MR images were scored by an experienced scorer who was blinded to clinical information. Of the 159 patients with AD, 48 consented to lumbar puncture for CSF biomarker testing. CSF samples (5–15 ml) were centrifuged at 2000 × *g* for 10 min and stored at −80°C, according to a previously established protocol. The levels of Aβ_42_, phosphorylated tau (p‐tau), and total tau (t‐tau), as well as the Aβ_42_/Aβ_40_ ratios in the CSF, were measured using enzyme‐linked immunosorbent assays.^26^

### 2.5 *APOE* genotyping

Of the 159 patients with AD, 10 refused to provide their blood samples. Thus, we assessed *APOE* genotyping in 149 patients with AD. *APOE* genotyping was performed on DNA extracted from a 10 ml blood sample using previously established protocols.^27^ The measurements were performed in a blinded manner.

### 2.6 Statistical analysis

Data are expressed as mean ± standard deviation, median or percentages. The Kolmogorov–Smirnov test was used to determine the normality of the data. Student's *t*‐tests and Chi‐square tests were used to assess the difference between two variable groups when the sample data were normally distributed; otherwise, the non‐parametric test was used. The covariance analysis was used to compare OCT measurements between AD patients and HCs, with age, sex, IOP, BCVA, and axial length (AL) taken as covariates. Similar analyses were performed for comparisons across AD subgroups and each subgroup versus HCs. We evaluated the correlation of OCT parameters with cognitive function, brain atrophy, CSF biomarkers, and *APOE* genotypes by using Pearson correlation analysis. Statistical significance was set at *p* < 0.05. All statistical analyses were performed using SPSS Statistics, version 25.0 (IBM Corp.).

### 2.7 The performance evaluation of different models

OCT variables with a significant *p*‐value at the above statistical analyses were incorporated into the final diagnosis model. We evaluated the performance of six common machine learning algorithms, including extreme gradient boosting (XGBoost), Light Gradient Boosting Machine (Light GBM), k‐nearest neighbor, Random Forest, Gradient Boost, and Adaptive Boosting (AdaBoost). We randomly selected 70% of the dataset to obtain the models and used the remaining 30% of the dataset to test the performance of the models. Four criteria were used to evaluate the performance of each model, including the accuracy (ACC), the area under the curve (AUC), f1 score, and recall. We used fivefold cross‐validation to validate the stability of these constructed models, and the accumulated ACC, AUC, f1‐score, and recall values were used as the metrics for evaluation. All the construction processes of prediction models were based on *Python* programming language (version 3.7).

## 3 RESULTS

### 3.1 Characteristics of study participants

We included 159 patients with AD and 299 HCs in the final analysis. We found no statistically significant differences in age, sex, or ophthalmologic parameters (IOP, BCVA, and AL). The AD group had a shorter mean duration of education (*p* < 0.001) and lower mini‐mental state examination (MMSE) scores (*p* < 0.001) than the HCs group (Table 1).

**TABLE 1:** Demographic characteristics of patients with AD and healthy controls

Variables	AD (*n* = 159)	HCs (*n* = 299)	*p* Value
Demographics			
Age, y, mean (SD)^a^	63.03 (9.06)	61.55 (8.92)	0.093
Sex, (m/f)^b^	47/112	78/221	0.427
Education, y, mean (SD)^a^	9.24 (4.46)	11.12 (4.55)	<0.001***
Disease duration, mean (SD)	2.76 (1.99)	NA	NA
Neuropsychological assessment, Mean (SD)			
MMSE^c^	15.14 (7.05)	23.64 (3.59)	<0.001***
MoCA	10.04 (6.07)	NA	NA
CDR	1.14 (0.81)	NA	NA
Ophthalmologic, Mean (SD)			
IOP (mm Hg)^a^	15.74 (2.76)	15.37 (2.75)	0.169
BCVA^a^	1.20 (0.25)	1.21 (0.26)	0.862
AL^a^	22.90 (0.93)	23.02 (0.86)	0.194

Abbreviations: AD, Alzheimer's disease; AL, axial length; BCVA, best‐corrected visual acuity; CDR, the global Clinical Dementia Rating; HCs, healthy controls; IOP, intraocular pressure; MMSE, Mini‐Mental State Examination; MoCA, Montreal Cognitive Assessment; SD, Standard deviation; y, years.

*Note*: Significant results in bold. *** *p* < 0.001.

^a^ Independent‐samples *t*‐test.

^b^ χ2 test.

^c^ Mann–Whitney U test.

### 3.2 HD‐OCT measures in AD and healthy controls

**FIGURE 3:**
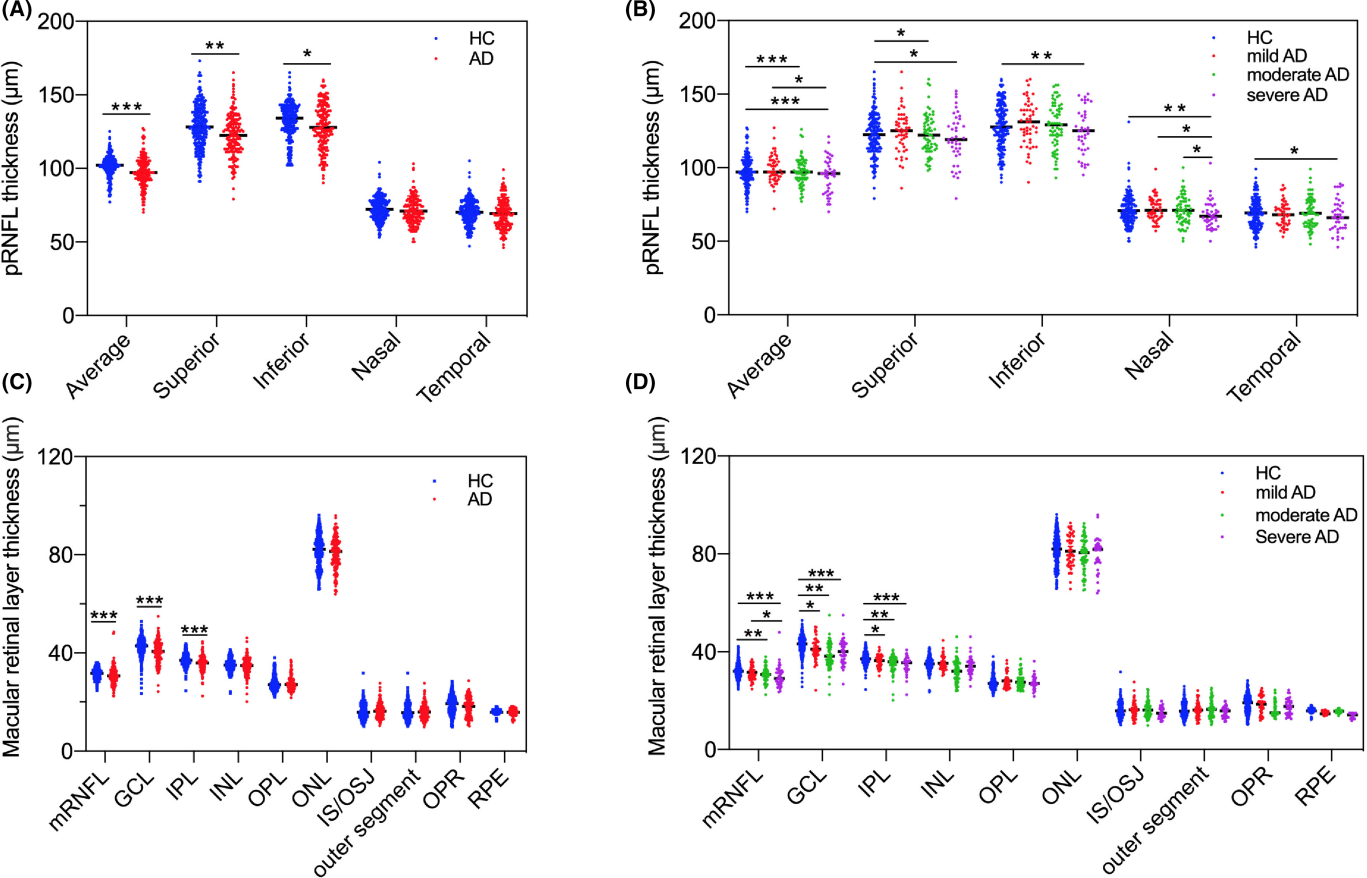
Comparison of OCT measures among (A), HCs and AD for pRNFL thickness; (B), subgroups of AD for pRNFL thickness; (C), HCs and AD for macular retinal layer thickness; (D), subgroups of AD for macular retinal layer thickness. The average, superior, and inferior quadrant pRNFL, and mRNFL, GCL, and IPL thicknesses were all significantly decreased in the AD group compared to the HCs group. In addition, GCL and IPL were significantly lower early in the mild AD group. * *p* < 0.05; ** *p* < 0.01; *** *p* < 0.001. OCT, optical coherence tomography; HCs, healthy controls; AD, Alzheimer's disease; pRNFL, peripapillary retinal nerve fiber layer; GCL, ganglion cell layer; IPL, inner plexiform layer.

After adjusting for potential confounders, the average, superior, and inferior quadrant pRNFL (Figure 3A), mRNFL, GCL, and IPL (Figure 3C) thicknesses were all significantly decreased in the AD group compared to the HCs group. In addition, AD group had significantly lower MRT compared to the HCs group (273.98 ± 12.53 μm vs. 283.20 ± 12.49 μm, *p* < 0.001) and lower TMV (9.87 ± 0.45 mm^3^ vs. 10.20 ± 0.45 mm^3^, *p* < 0.001).

The AD patients were divided into the following three different severity subgroups based on clinical dementia rating (CDR) scores: mild (*n* = 51, CDR = 0.5), moderate (*n* = 67, CDR = 1), and severe (*n* = 41, CDR = 2) groups. The OCT measurements were compared between each AD subgroup and HCs. The results showed that GCL (*p* = 0.020) and IPL (*p* = 0.044) were significantly lower early in the mild AD group (Figure 1D). In addition, compared to the HCs group, the average (*p* < 0.001), superior (*p* = 0.015) quadrant pRNFL thickness (Figure 3B), mRNFL (*p* = 0.004) thickness (Figure 3D), MRT (*p* < 0.001) and TMV (*p* < 0.001) values in the moderate AD group were significantly decreased, and the inferior (*p* = 0.003), nasal (*p* = 0.003), and temporal (*p* = 0.022) quadrant pRNFL thickness (Figure 3B) were significantly decreased only in the severe AD group.

### 3.3 Correlation between OCT measures and cognitive performance

**FIGURE 4:**
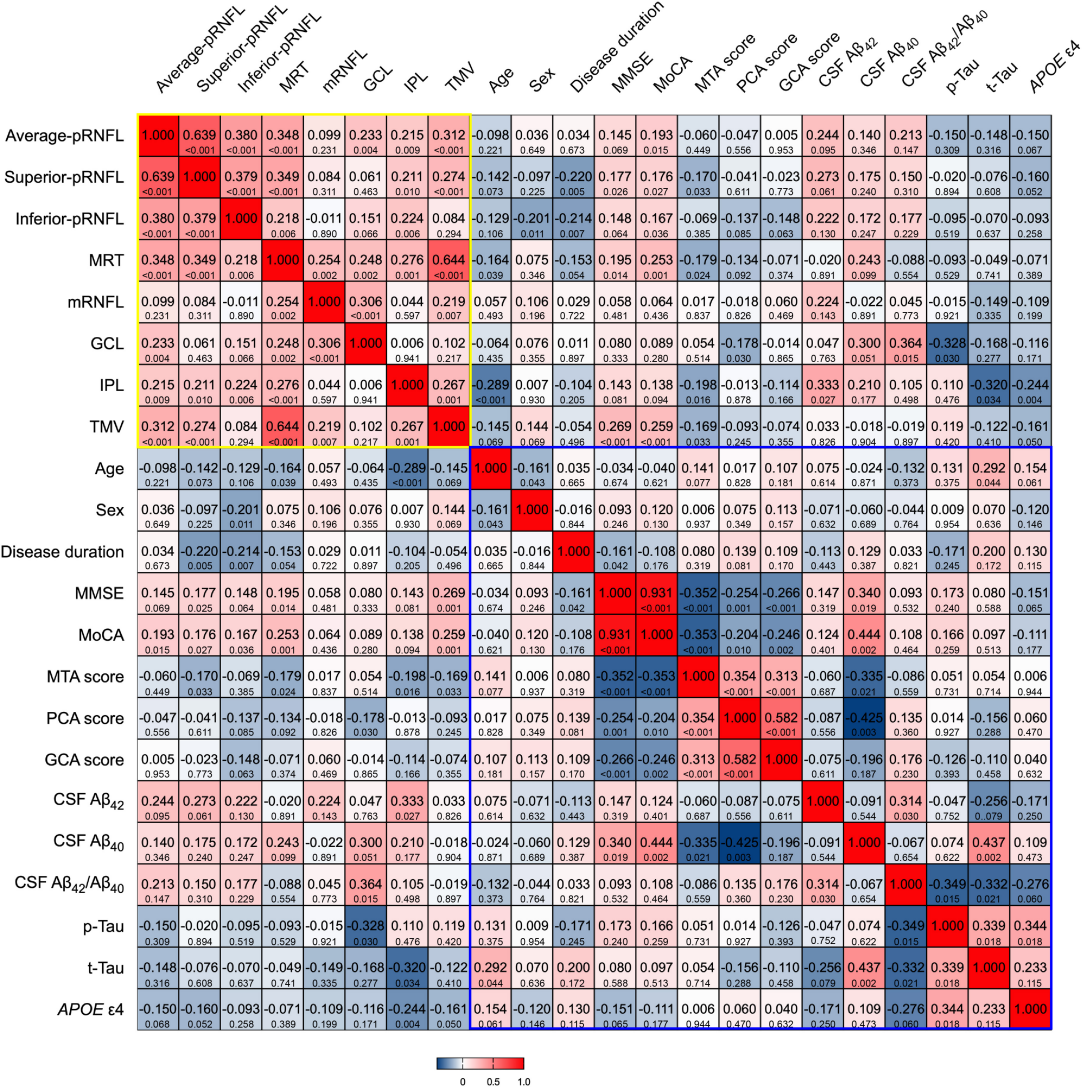
Heat map of correlation between OCT measures and clinical parameters, AD biomarkers in patients with AD. There was a significantly positive correlation between TMV and MMSE, MoCA scores. In addition, IPL thickness was correlated negatively with the MTA score, and the GCL thickness was positively correlated with CSF Aβ_42_/Aβ_40_ and negatively associated with p‐tau level. The top row of each cell is Pearson's correlation coefficients, and the bottom row is the corresponding *p*‐value. The yellow and blue boxes represented the correlations among OCT parameters, and among clinical parameters, respectively. OCT, optical coherence tomography; AD, Alzheimer's disease; TMV, total macular volume; MMSE, Mini‐Mental State Examination; MoCA, Montreal Cognitive Assessment; IPL, inner plexiform layer; MTA, medial temporal lobe atrophy; GCL, ganglion cell layer; CSF, cerebrospinal fluid.

We examined the correlations between the OCT measures and cognitive function assessed by MMSE and Montreal cognitive assessment (MoCA) scores in patients with AD. After adjusting for potential confounders, superior quadrant pRNFL thickness (*β* = 0.177, *p* = 0.026), MRT (*β* = 0.195, *p* = 0.014), and TMV (*β* = 0.269, *p* < 0.001) were positively associated with MMSE scores. Moreover, the average (*β* = 0.193, *p* = 0.015), superior (*β* = 0.176, *p* = 0.027), inferior (*β* = 0.167, *p* = 0.036) quadrant pRNFL thickness, MRT (*β* = 0.253, *p* = 0.001), and TMV (*β* = 0.259, *p* < 0.001) were positively associated with MoCA scores (Figure 4).

### 3.4 Correlations between OCT measures, MRI, and CSF biomarkers

Correlations between OCT measures and brain atrophy assessed by MTA, PCA, and GCA scores were also analyzed. After adjusting for potential confounders, we observed a significant inverse association of the MTA score with the superior quadrant pRNFL thickness (*β* = −0.170, *p* = 0.033), MRT (*β* = −0.179, *p* = 0.024), IPL thickness (*β* = −0.198, *p* = 0.016), and TMV (*β* = −0.169, *p* = 0.033) in AD patients. Moreover, the GCL thickness correlated negatively with the PCA score (*β* = −0.178, *p* = 0.030). No significant correlations were observed between any OCT measure and the GCA score (Figure 4).

Among 48 AD patients with available CSF samples, 37 exhibited reduced Aβ_42_ or Aβ_42_/Aβ_40_ levels, and 29 had a concomitant increase in p‐tau protein levels. Correlation analyses between OCT measurements and AD biomarker results showed that the GCL thickness was positively correlated with CSF Aβ_42_/Aβ_40_ (*β* = 0.364; *p* = 0.015) and negatively associated with CSF p‐tau concentrations (*β* = −0.328; *p* = 0.030) in the AD group. The IPL thickness was positively correlated with CSF Aβ_42_ concentrations (*β* = 0.333; *p* = 0.027) and negatively associated with CSF t‐tau concentrations (*β* = −0.320; *p* = 0.034) in the AD group. However, no association was found between CSF biomarkers and pRNFL thickness (Figure 4).

### 3.5 Correlations between OCT measures and *APOE* genotypes

The results showed that 66 (44.30%) patients with AD were *APOE* ε4‐noncarriers (ε2/ε3, ε3/ε3), whereas 83 (55.70%) patients with AD were *APOE* ε4‐carriers (ε2/ε4, ε3/ε4, ε4/ε4). We divided patients with AD into two groups based on *APOE* genotypes: the *APOE* ε4‐noncarrier and *APOE* ε4‐carrier groups. All retinal parameters were decreased in the *APOE* ε4‐carrier group compared to the *APOE* ε4‐noncarrier group, though this difference was only significant in IPL thickness (*p* = 0.004) (Figure 4).

### 3.6 The performance of different models in AD prediction

**FIGURE 5:**
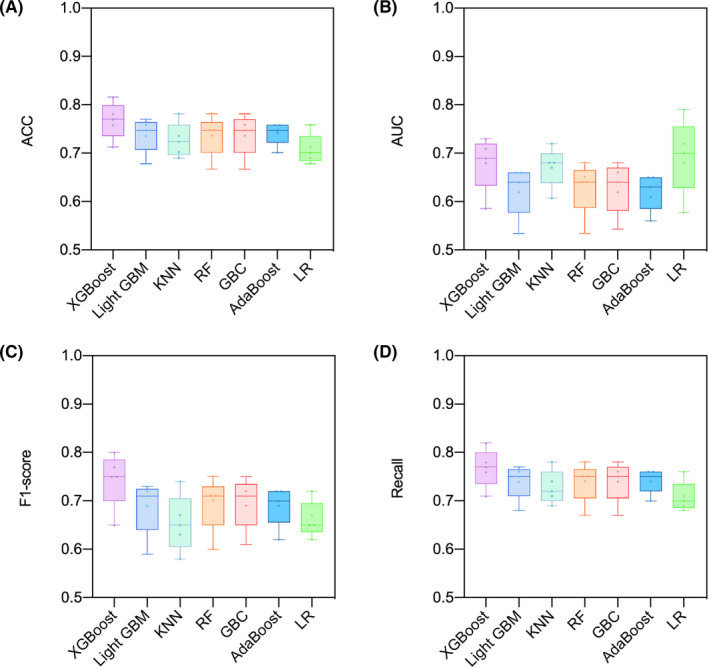
The fivefold cross‐validated results of different models in the testing set. The result showed that the XGBoost algorithm was superior to traditional Logistic Regression and the other five machine learning algorithms. (A), accuracy; (B), the area under the curve; (C), f1‐score; (D), Recall. Abbreviation: KNN, k‐nearest neighbor; RF, Random Forest; GBC, Gradient Boost Classifier; LR, Logistic Regression.

**FIGURE 6:**
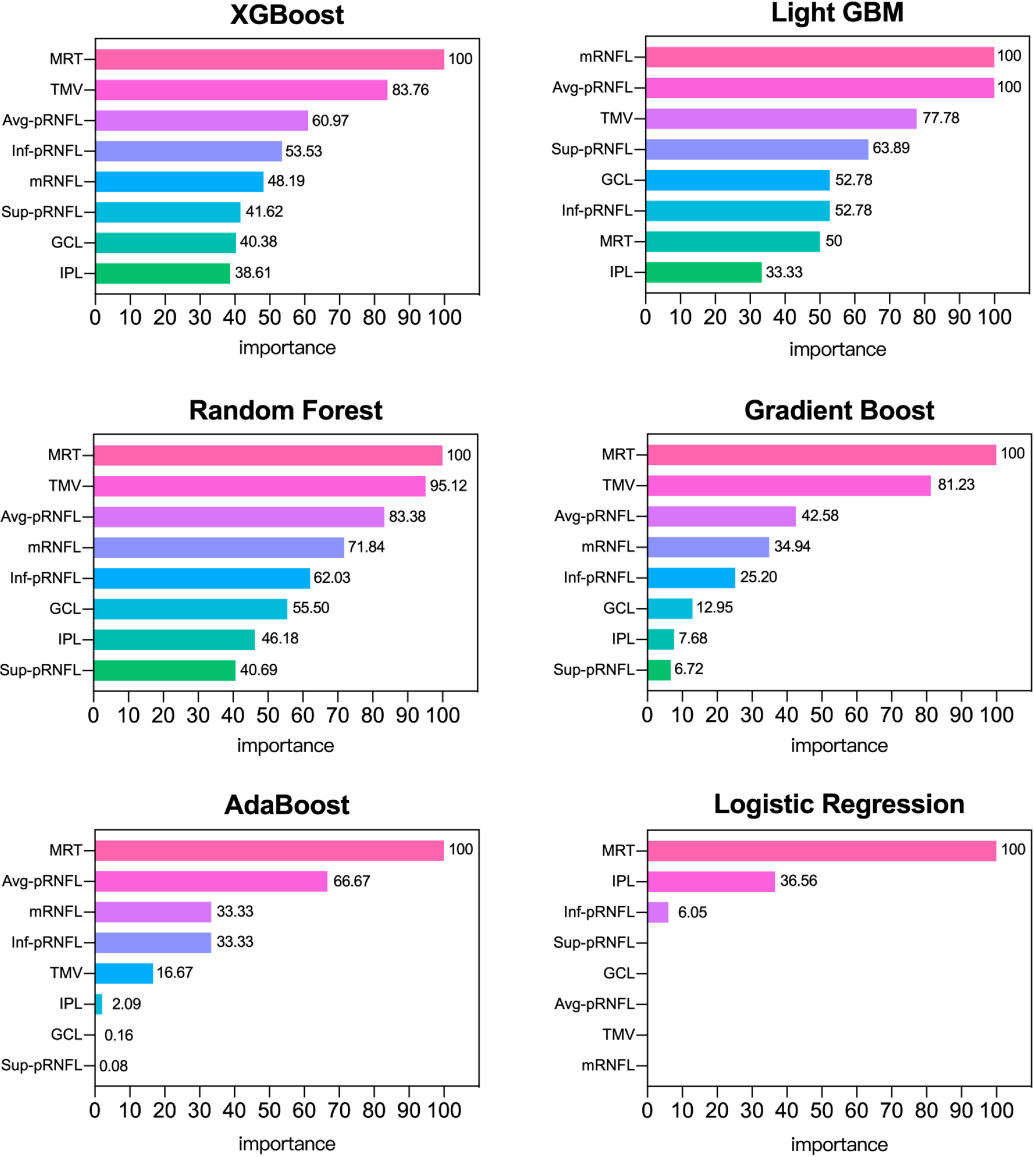
The weight plots of different variables from five machine learning models. The length of the bar indicates the importance of the variable. To facilitate a more intuitive comparison, this most important variable was taken as the baseline reference value, and other variables were presented as percentages of the reference value. There is no weight plot from KNN, which is a clustering algorithm, and thus is not amenable to linear fitting.

Three pRNFL (average, superior, and inferior pRNFL thickness) and five macular (MRT, TMV, mRNFL, GCL, and IPL thickness) variables showed a significant decrease in patients with AD. All participants were randomly split into training (111 AD patients and 209 HCs) and testing (48 AD patients and 90 HCs) sets. The results revealed that all six machine learning algorithms were superior to the traditional Logistic Regression. Moreover, the XGBoost algorithm exhibited the best diagnostic performance in both the training and testing sets. In the testing set, the evaluated results of the XGBoost algorithm were ACC (0.74), AUC (0.69), f1 score (0.70), and recall (0.74) (Table 2). To verify the stability of these constructed models, the fivefold cross‐validation was conducted, and the accumulated ACC, AUC, f1‐score, and recall values were used as the indicators for evaluation. The cross‐validation results of these models showed that the XGBoost algorithm was superior to traditional Logistic Regression and the other five machine learning algorithms (Figure 5). The most important variable in the five models (XGBoost, Random Forest, Gradient Boost, AdaBoost, and Logistic Regression) was MRT, which demonstrated absolute superiority compared to the other variables. In contrast, the mRNFL thickness was the main influential factor in the Light GBM model (Figure 6).

**TABLE 2:** The performance of different models in AD prediction

Models	ACC	AUC	f1 score	recall
Training Set				
XGBoost	0.94	0.91	0.94	0.94
Light GBM	0.77	0.93	0.73	0.77
KNN	0.71	0.74	0.62	0.71
Random Forest	0.90	0.86	0.90	0.90
Gradient Boost	0.76	0.63	0.71	0.76
AdaBoost	0.76	0.76	0.70	0.76
Logistic Regression	0.69	0.53	0.58	0.69
Testing Set				
XGBoost	0.74	0.69	0.70	0.74
Light GBM	0.72	0.71	0.68	0.72
KNN	0.70	0.68	0.60	0.70
Random Forest	0.72	0.73	0.68	0.72
Gradient Boost	0.70	0.75	0.61	0.70
AdaBoost	0.73	0.69	0.68	0.73
Logistic Regression	0.67	0.52	0.53	0.67

Abbreviations: ACC, accuracy; AD, Alzheimer's disease; AdaBoost, Adaptive boosting; AUC, the area under the curve; GCL, ganglion cell layer; IPL, inner plexiform layer; KNN, k‐nearest neighbor; Light GBM, Light Gradient Boosting Machine; mRNFL, macular retinal nerve fiber layer; MRT, macular retina thickness; TMV, total macular volume; XGBoost, extreme gradient boosting.

*Note*: Three pRNFL variables (Average, Superior, Inferior pRNFL) and five macular variables (MRT, TMV, mRNFL, GCL, IPL) were incorporated into the final prediction model. Machine‐learning methods included XGB Classifier, Light GBM, KNN, Random Forest, Gradient Boosting Classifier, and AdaBoost Classifier. All participants were randomly split into the training set and testing set according to a 7:3 ratio.

## 4 DISCUSSION

This study revealed that OCT measurements were significantly correlated with MMSE and MoCA scores indicative of cognitive function, MTA and PCA scores indicative of cerebral atrophy, tau and Aβ levels in the CSF, and *APOE* genotypes in patients with AD. In addition, we found that the XGBoost algorithm showed the best diagnostic performance for AD by comparing six common machine learning algorithms' performances.

Consistent with previous studies, the present study found pRNFL thinning in the superior and inferior quadrants.^28^, ^29^ Axons from the superior and the inferior retina project to the cuneal gyrus and lingual gyrus, where deposition of Aβ plaques and neurofibrillary tangles have been well‐reported.^9^, ^30^ Retinal ganglion cells (RGCs) and their fibers are located in the macular area, a region in which progressive loss and degeneration of RGCs have been detected in AD patients.^31^ The retinal Aβ deposits are more concentrated in the inner macular layer (mRNFL, GCL, and IPL), leading to the thinning of these layers.^31^ It could also be due to the degeneration of RGCs and their axons or the retrograde trans‐synaptic degeneration of the RGC layer and its axons.^32^ Furthermore, the degeneration of RGC dendrites confined to the retinal IPL preceded cell loss in an AD mouse model,^33^ indicating that the inner retinal layer may prove to be a potential site of interest for early AD detection.

We found that retinal measures were positively associated with MMSE and MoCA scores. This suggests that OCT parameters correlate with cognitive function in patients with AD. Oktem et al.^34^ also found there was a significant correlation between RNFL thickness and MMSE scores. In addition, Iseri et al.^35^ reported a relationship between TMV and MMSE scores in patients with AD. It is well known that MMSE and MoCA scores dropped as AD progressed. Therefore, the correlation between OCT measurements and MMSE and MoCA scores supports that retinal thickness was affected by disease severity. Hence, OCT might be a useful tool for monitoring the disease progression of AD and evaluating the efficacy of new therapeutic strategies.

An association was also detected between some OCT measures and MTA and PCA scores. This study demonstrated that both axonal and ganglionic cell body losses in the retina were associated with MTA scores and that the GCL thickness was correlated with the PCA score. The hippocampus is known to be involved in the pathogenesis of neurodegenerative diseases, including AD,^36^ and the posterior cortex is involved in visual processing; consequently, retrograde retinal atrophy may occur in AD.^37^ The cingulate cortex is the central hub of cognitive brain networks, particularly the posterior cingulate cortex, which is important for processing episodic memory tasks.^38^ The findings from recent studies further suggest that the hippocampus and cingulate cortex play key roles in the neurodegenerative processes emblematic of AD. However, we found no significant associations between OCT measures and GCA scores. Again, this is likely because of the high proportion of mild‐to‐moderate AD patients in our dataset (74.21%). The global brain structures of such patients are often preserved; therefore, few patients with AD might have developed whole‐brain alterations in our study.

Our study showed that GCL thickness was positively correlated with CSF Aβ_42_/ Aβ_40_ and negatively correlated with CSF p‐tau levels. Additionally, IPL thickness was positively correlated with CSF Aβ_42_ levels and negatively correlated with CSF t‐tau levels. There are few reports on the association between retinal thickness and CSF biomarkers. A study found that neither pRNFL nor macular thickness was associated with CSF biomarkers.^16^ However, their study sample size was small, including only 15 patients with AD. In postmortem studies, Aβ deposits and p‐tau protein have been observed in patients with AD.^39^ We hypothesize that the levels of Aβ and tau in the retina mirror those in the brain. A lower CSF Aβ_42_ and a higher tau concentration reflect a higher Aβ and tau burden in the retina and brain. Thus, the retinal pathophysiological changes could eventually lead to decreased retinal thickness.

We found that all retinal parameters were decreased in the *APOE* ε4‐carrier group compared to the *APOE* ε4‐noncarrier group, although this difference was only significant in terms of IPL thickness. In previous studies, patients with AD carrying *APOE* ε4 have been shown to exhibit increased apoptosis and loss of synaptic integrity.^40^ In a mouse model, *APOE* ε4 was also associated with reduced microvascular density and neocortical cerebral blood flow.^41^ Retinal capillary density is associated with brain perfusion and is reduced in early AD.^42^ Therefore, we speculate that retinal thinning is followed by a reduction in microvascular perfusion of the retina. The IPL, where bipolar and amacrine cells form a synapse with the ganglion cells, was rich in cholinergic synapses and could release acetylcholine. The IPL thickness was significantly decreased in the *APOE* ε4‐carrier group, possibly due to cholinergic dysfunction in the IPL, as described in previous studies.^43^

In this study, we presented a machine learning model to diagnose AD. The primary objective of our study was to develop a diagnostic prediction model for AD by combining OCT measures and machine learning. Eight quantitative OCT features were screened for inclusion in the final diagnostic models. Obtaining OCT features was inexpensive, noninvasive, and convenient when compared to traditional diagnostic tools (MRI, CSF, or PET). Additionally, our study revealed that the XGBoost algorithm exhibited the best diagnosis performance for AD, including the highest ACC, f1 score, and recall both for the training and testing sets. The superiority of the XGBoost algorithm in diagnostic performance was validated for these evaluation indicators, including ACC, f1 score, and recall by using fivefold cross‐validation. The XGBoost algorithm based on OCT measurements was superior to traditional logistic regression and other five machine learning algorithms and exhibited certain potential in the diagnosis of AD. Additionally, we found that all six machine learning algorithms were superior to the traditional Logistic Regression. This confirmed that machine learning had a wide range of application prospects in clinical practice. Because of its ability to detect complex patterns and construct models, it was available to assist in the clinical diagnosis of AD, and thus, achieve the aim of the early invention and optimizing treatment strategies. Further studies are required to validate these findings.

This study had some limitations. Since OCT measurements and neuropsychological assessments require patients' cooperation, patients with more advanced stage AD (CDR = 3) could not be included in our study. Additionally, this was a cross‐sectional study; therefore, we cannot conclude retinal changes over time. However, we compared OCT measurements among patient subgroups according to disease severity and HCs groups, which roughly simulates the process of retinal changes over time. Moreover, MRI and CSF analyses were not available for the HC group.

In conclusion, this large‐scale study confirmed that retinal structure was significantly altered in patients with AD and that OCT measurements correlated with cognitive function, MRI findings indicative of cerebral atrophy, tau and Aβ levels in the CSF, and *APOE* genotypes. In addition, we developed an optimal machine learning algorithm to assist the clinical diagnosis of AD. Overall, this study confirmed that OCT measurements combined with machine learning could be useful for AD diagnosis.

## AUTHOR CONTRIBUTIONS

XW and BJ designed the experiment and analyzed, interpreted the data, and wrote the manuscript. XW, BJ, HL, YQW, XLH, YZ, SZZ, QX, XXL, YFZ, HJ, JLW, JFG, XXY, and BST collected participants' data and discussed the results. BX and HZX performed all participants' OCT images scan. XLJ and RCZ segmented and calculated the macular single retinal layer thickness of all scans. LS edited the manuscript.

## CONFLICT OF INTEREST

The authors have no competing interests to declare.

## Data Availability

All data that support the findings of the current study are available from the corresponding authors upon reasonable request.
